# Circulating immune cells exhibit distinct traits linked to metastatic burden in breast cancer

**DOI:** 10.1186/s13058-025-01982-2

**Published:** 2025-05-08

**Authors:** S. Mangiola, R. Brown, C. Zhan, J. Berthelet, S. Guleria, C. Liyanage, S. Ostrouska, J. Wilcox, M. Merdas, P. Fuge-Larsen, C. Bell, J. Schröder, L. A. Mielke, J. M. Mariadason, S. Chang-Hao Tsao, Y. Chen, V. K. Yadav, S. Vodala, R. L. Anderson, D. Merino, A. Behren, B. Yeo, A. T. Papenfuss, B. Pal

**Affiliations:** 1 The Walter and Eliza Hall Institute of Medical Research, Parkville, VIC 3052 Australia; 2 Department of Medical Biology, University of Melbourne, Parkville, VIC 3052 Australia; 3 South Australian immunoGENomics Cancer Institute, Adelaide, SA 5005 Australia; 4 School of Cancer Medicine, La Trobe University, Bundoora, VIC 3086 Australia; 5 Olivia Newton-John Cancer Research Institute, Heidelberg, VIC 3084 Australia; 6 Peter Doherty Institute for Infection and Immunity, Parkville, VIC 3052 Australia; 7 The University of Melbourne, Parkville, VIC 3052 Australia; 8 Austin Health, Heidelberg, VIC 3084 Australia; 9 Rutgers New Jersey Medical School, Newark, NJ USA; 10 Boehringer Ingelheim Pharmaceuticals Inc, Ridgefield, USA; 11 La Trobe Institute for Molecular Science, La Trobe University, Bundoora, VIC 3086 Australia

**Keywords:** PBMCs, Breast cancer, Metastasis, Single-cell RNA sequencing

## Abstract

**Background:**

Circulating immune cells play a crucial role in the anti-tumour immune response, yet the systemic immune system in metastatic breast cancers is not fully characterised. Investigating the cellular and molecular changes in peripheral blood mononuclear cells (PBMCs) from breast cancer patients could elucidate the role of circulating immune cells in metastasis and aid in identifying biomarkers for disease burden and progression.

**Methods:**

In this study, we characterised the systemic immune landscape associated with varying levels of metastatic burden by analysing the single-cell transcriptomes of PBMCs from breast cancer patients and healthy controls. Our research focused on identifying changes in immune cell composition, transcriptional programs, and immune-cell communication networks linked to metastatic burden. Additionally, we compared these PBMC features onto a single-cell atlas of primary breast tumours to study corresponding traits in tumour-infiltrating immune cells.

**Results:**

In metastatic breast cancer, PBMCs exhibit a significant downregulation of the adaptive immune system and a decreased number and activity of unconventional T cells, such as γδ T cells. Additionally, metastatic burden is associated with impaired cell communication pathways involved in immunomodulatory functions. We also identified a gene signature derived from myeloid cells shared between tumour immune infiltrates and circulating immune cells in breast cancer patients.

**Conclusions:**

Our study provides a comprehensive single-cell molecular profile of the peripheral immune system in breast cancer, offering a valuable resource for understanding metastatic disease in terms of tumour burden. By identifying immune traits linked to metastasis, we have unveiled potential new biomarkers of metastatic disease.

**Supplementary Information:**

The online version contains supplementary material available at 10.1186/s13058-025-01982-2.

## Introduction

Metastatic breast cancer (MBC) is generally incurable, with 10–20% of patients developing fatal metastatic lesions at multiple sites, including lung, liver, bone and brain [1, 2]. Breast cancer can metastasise with variable volumes of organ involvement [3]. The timely detection of widespread distant metastases remains a significant challenge in the treatment and care of breast cancer patients. Unlike existing approaches, including biopsies and imaging, blood-based detection methods minimise invasiveness while reducing sample bias and increasing sensitivity using molecular markers. In recent years, efforts have been made to study blood-based biomarkers for cancer detection through qualitative and quantitative analyses of circulating tumour cells (CTCs) and circulating tumour DNA (ctDNA) [4–6]. While CTCs and ctDNA present promising non-invasive methods for cancer diagnosis and monitoring, their clinical application is hindered by challenges in detection and isolation due to their low abundance in the bloodstream, affecting the accuracy and reliability of the results. Although ctDNA can provide valuable information on genetic mutations and tumour dynamics, it may not capture other crucial aspects of the tumour, such as protein expression or interactions within the microenvironment [7, 8]. Further research into other blood components, such as PBMCs, is essential to overcome some of these limitations of blood-based liquid biopsy methods and fully realise their potential in clinical practice.

Peripheral blood mononuclear cells (PBMCs), a component of the circulating immune cells, are considered the first line of defence against cancer [9, 10]. These cells are vital in responding to and being recruited to primary and metastatic tumour sites. Recent studies have shown that PBMCs contain cancer-specific biomarkers for the detection and prognosis of some cancer types, such as advanced renal cell carcinoma, non-small cell lung cancer and hepatocellular carcinoma [11–14]. Thus, exploring the diverse cellular composition and transcriptomic changes of PBMCs associated with metastasis could establish a molecular foundation and predictive biomarkers for metastatic progression in cancer patients. In breast cancer, PBMCs of triple-negative breast cancer (TNBC) patients show compositional changes in immunosuppressive cell types and enrichment of a predictive gene signature (CD163, CXCR4, and THBS1) for relapse-free survival [15]. PBMC transcriptome profiles have also been used to subclassify breast cancer patients based on lymphocyte or neutrophil-polarised immune responses [16]. Whether these compositional and transcriptomic changes in PBMCs can be exploited as novel biomarkers of metastatic disease or hard-to-biopsy metastatic sites in breast cancer remains to be investigated.

Herein, we used single-cell RNA sequencing to investigate PBMCs isolated from ten patients diagnosed with metastatic breast cancer, with five classified as low metastatic burden patients and the other five with more extensive metastatic burden. By analysing the immune profiles of these patients, we aimed to establish a reliable molecular biomarker for identifying patients with low-volume metastasis amendable to localised therapies. Our study revealed a strong relationship between the single-cell transcriptomes of circulating immune cells and the extent of metastatic spread of breast cancer, thereby revealing unique biomarkers of metastatic burden. Our findings could assist in designing improved detection and treatment strategies against aggressive breast cancer and may have significant implications for personalised cancer care and treatment decision-making.

## Material and methods

### Peripheral blood mononuclear cells (PBMCs) isolation from blood samples

The protocol allows the separation of plasma, PBMCs, and granulocytes from a single blood sample. Blood collected in K2-EDTA tubes was layered onto Ficoll-Paque (GEHE17-1440–03, Biostrategy PTY) for density gradient centrifugation at 1000 g at 21 °C for 25 min. Plasma was aspirated, centrifuged at 300 g (4 °C, 7 min), and snap-frozen. The PBMC layer (buffy coat) was carefully collected, washed with PBS, centrifuged at 300 g (4 °C, 7 min), and cryopreserved in 10% DMSO in FBS. Granulocyte pellets underwent red blood cell lysis using eBioscience™ 10X RBC Lysis Buffer (diluted to 1X with sterile ddH2O), followed by PBS washes and cryopreservation.

### 10 X Genomics Chromium library construction and sequencing

A 10 X Genomics Chromium machine was used for single-cell capture and cDNA preparation according to the manufacturer’s Single Cell 3’ Protocol. The silane magnetic beads and Solid Phase Reversible Immobilization (SPRI) beads were used to clean up the GEM reaction mixture, and the barcoded cDNA was then amplified in a PCR step. The optimal cDNA amplicon size was achieved using the Covaris machine before library construction. The P7 and R2 primers were added during the GEM incubation, and the P5 and R1 during library construction via end repair, A-tailing, adaptor ligation and PCR. The final libraries contain the P5 and P7 primers used in Illumina bridge amplification. Sequencing was carried out on an Illumina Nextseq 500.

### Single-cell read mapping and quality control

The read information was converted to FASTQ files using bcl2fastq, mapped to the Hg38 3.0.0 genome and quantified using CellRanger [17] (version 7.0.0) with default parameters (–expect-cells = 7000). The gene counts were imported to a SingleCellExperiment object [18] using DropletUtils [19] and manipulated using tidyomics [20]. Quality control and filtering were performed independently for each sample. The empty droplets were identified using barcodeRanks [19] below the inflection point. The mitochondrial gene names were downloaded using AnnotationDbi (mapIds) and EnsDb.Hsapiens.v86. Scuttle [21] was used to calculate cell quality metrics (perCellQCMetrics) using default parameters. The function isOutlier was used with default parameters to label outlier cells based on the fraction of mitochondrial transcription and ribosomal genes. For subsequent quality control and analyses, the data was converted to a Seurat container, and tidyseurat [22] was used for data manipulation. DoubletFinder (v3) [23] was used with default parameters to identify and filter out doublets.

### Label transfer for healthy versus cancer analyses

For the healthy control (Supp. Table 1), we used PBMC data from the CELLxGENE repository (https://cellxgene.cziscience.com) [24–28] and the public sources SCP345 (singlecell.broadinstitute.org), SCP424 [29], SRR7244582 [30]. We selected specimens of healthy status from female blood (doublechecked for the XIST gene expression > > 0). All samples were subject to quality control and filtering. Considering the heterogeneous nature of the public data within CELLxGENE, we employed a label transfer approach to compare cancer with healthy data. Seurat software was used to perform Azimuth label transfer with the PBMC reference [31], with default parameters. To void under-heterogeneous samples, we further filtered samples with at least 15 detected cell types, and then we filtered datasets with at least one sample.

### Normalisation, integration, and annotation for high versus low burden analyses

We identified variable genes within each sample using Seurat [32]. We estimated the cell cycle phase using CellCycleScoring. Using principal component analysis (PCA) and UMAP dimensionality reduction, we checked that the cell cycle was not a major confounder for cell distribution and grouping. We scaled counts cell-wise and removed the unwanted variation of residual mitochondrial and ribosomal content using the SCT transform [32]. The unwanted sample-wise technical variation was removed using the anchor-based integration implemented in Seurat [33], using SI-GA-E5 as the reference sample (as it was of excellent quality according to analyses and sequencing output). A nearest-neighbour graph is calculated from adjusted counts, and cells are clustered [34]. For the first instance classification, we transferred cell-type annotation from the reference PBMC Azimuth dataset using Seurat [35].

We then isolated the macro clusters lymphoid, myeloid, and b-cells for independent integration, clustering, and marker gene selection. For each macro cluster, Seurat was used to identify cluster marker genes (FindAllMarkers, with the parameters only.pos = TRUE, min.pct = 0.25, thresh.use = 0.25). The curated cell annotation was based on a consensus between the identified markers and the label transfer described above (using pbmc_multimodal as the reference). The classification of the gamma delta population was refined using an ad hoc signature [36]. This signature included markers for gamma delta T cells vd1 and vd2. The gamma delta cells were further isolated, re-clustered (3 clusters found), and annotated with the ad hoc signature. Based on the cluster gene transcription abundance. The annotated macro clusters were then merged into a unique dataset.

### Differential tissue composition

The sccomp method was used for differential composition and variability analyses [37]. Predictive posterior intervals were checked after fit to assess the model’s descriptive adequacy to the data. sccomp identified outlier observations (cell type abundance) and dropped them from the hypothesis testing.For the healthy vs cancer analyses the unwanted study effect, completely confounded with the disease, was modelled as ~ 0 + cancer_high_burden + cancer_low_burden + healthy dataset1 + … + healthy_datasetN, and the contrasts were used to compare the disease states, i.e. cancer_vs_healthy = (cancer_high_burden + cancer_low_burden)/2 – (healthy_dataset1 + … + healthy_datasetN)/N.

### Differential gene transcription abundance

The differential analyses were performed on pseudo-bulk sample/cell-type pairs. The framework tidybulk [38] was used to perform the analyses based on the robust edgeR implementation [39]. The hypothesis tested log twofold changes bigger than 1 [40, 41]. The significant genes that included outliers were filtered out. The model, applied to each cell type, and hypothesis testing strategy was the same as for the composition analyses.

### Differential cell communication

The framework CellChat [42] was used to infer the transcriptional abundance of ligand-receptor pairs between cell types. The transcriptional abundance of ligand-receptor pairs between cell types was estimated for each sample independently (with default parameters). The centrality of communication axes was estimated. We calculated the mean across samples of the same metastatic profile (low or high burden) of the transcription score of ligand-receptor pairs between cell types. We then calculated the difference in the means to estimate the overall difference in communication. We confirmed the most significant communication changes at the gene count level. We created pseudobulk count aggregating cells at the sample/cell-type level. We selected the involved genes for each communication axis and tested the difference in pseudobulk transcription levels. Transcriptional levels were scaled for sample sequencing depth and sample-wise cell type abundance using TMM [43].

### Miscellaneous bioinformatics tools

We used the colour-deficiency-friendly code from dittoSeq [44]. For visualisation, we used mainly ggplot2 [45]. For heatmaps, we used tidyHeatmap [38, 46]. For data wrangling, we used the tidyverse software suite [47]. For pseudobulk differential transcript abundance analyses, we used tidybulk [38].

### Fluorescent multiplex-immunohistochemistry (mIHC)

Breast tumour sections were stained with antibodies using the Opal 6-plex kit for multiplex fluorescent IHC immunohistochemistry kit (NEL811001KT, Akoya Biosciences). Staining was performed manually per the manufacturer’s protocol, as previously described (79). The following antibodies were used: CD3 (SP7, Invitrogen, 1:150 dilution), TCRδ (H-41, Santa Cruz Biotech, 1:100 dilution), pan Cytokeratin (AE1/AE3 + 5D3, Abcam, 1:200 dilution), FGFBP2 (polyclonal, Invitrogen, 1:700 dilution), FoxP3 (236A/E7, Abcam, 1:300 dilution) and spectral DAPI (Akoya Biosciences). Whole slide scans at 10 × magnification were performed using the Perkin Elmer Vectra Imaging System to mark regions of interest and obtain 20 × magnification multispectral images (MSI) for analysis. To quantitatively analyse cell populations, a spectral library was created on InForm Analysis Software (Akoya Biosciences). Immune cell populations of interest were identified by each marker’s mean fluorescence intensity (MFI), and the algorithm was applied to all samples. Images were analysed on HALO (Indica Labs) using the Highplex v4.1.3 module for target cell phenotyping.

## Results

### Circulating immune cells show significant compositional and transcriptional shifts in cancer condition

PBMCs were isolated from the blood samples of 10 female breast cancer patients (Supp. Table 1) diagnosed with metastatic disease. We used the droplet-based 10X Chromium workflow to capture single cells and prepare single-cell RNA sequencing libraries for PBMCs. The sequencing data was generated from 60,501 cells with a median of 3,776 per sample. Across samples, cells had a median RNA read output from 629 to 4,110 (Supp. Figure S1A and Supp. Table 2). The rank of cells based on total RNA output (barcodeRanks [48]) followed a nominal curve for all samples (Supp. Figure S1A). We excluded a median of 3% of all cells (across samples) from analysis due to high mitochondrial transcripts and a median of 6.7% of all remaining cells across samples as doublets.

**Fig. 1:**
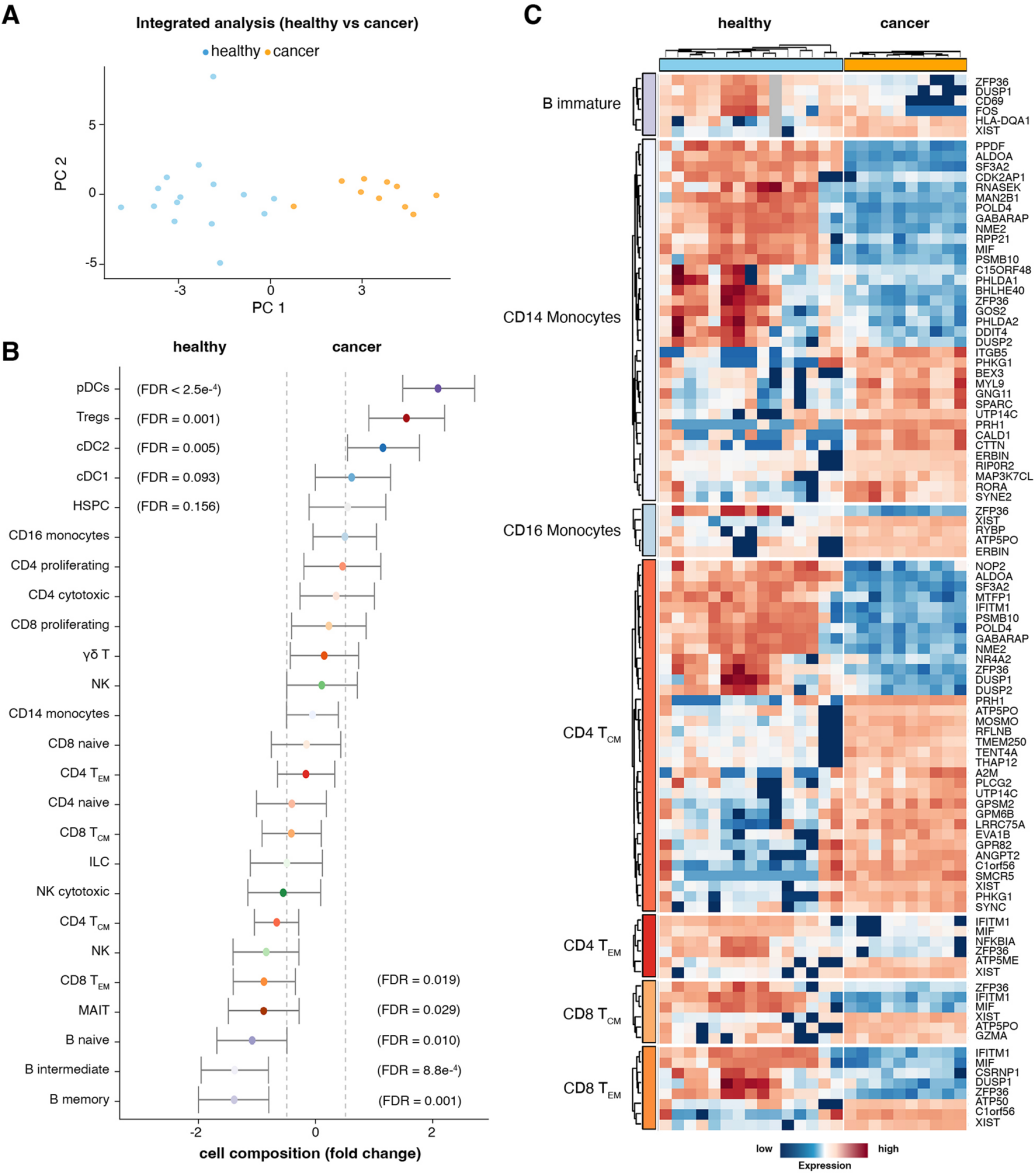
PBMCs show unique cell composition and phenotype. **A** PCA plot of the 25 pseudo-bulk transcriptomes, coloured by healthy (blue) and metastatic (orange) conditions. **B** Differential cell composition between healthy and cancer patients. Dots are coloured by cell type (also described in Fig. 2C). The right side of the plot contains cells that are enriched in cancer patients, while the cells on the left are enriched in healthy donors. The error bars represent the 95% credible interval for the estimates. The significantly enriched cell types are defined by having the 95% credible interval outside the −0.5/0.5 logit-fold-change interval. **C** Scaled transcription abundance of cell-type-specific pseudo-bulk samples for the top differentially abundant gene transcripts between healthy vs metastatic breast cancer (FDR < 0.01; log fold change > 2). pDC, Plasmacytoid dendritic cells; cDC, Conventional dendritic cells; Tregs, Regulatory T cells; HSPC, Hematopoietic stem and progenitor cells; NK, natural killer; MAIT, Mucosal-associated invariant T cells

To evaluate the extent to which immune blood features reflect the metastasis-associated inflammatory condition, we integrated both cancer and 15 publicly available single-cell RNA sequencing datasets of healthy donors (Supp. Table 1) [24–30] with a PBMC reference using Azimuth [31]. The principal component analysis of the healthy and cancer cohort, using pseudobulk representation of the single-cell data, separated samples based on clinical characteristics (healthy vs. cancer) (Fig. 1A). We performed compositional analyses using sccomp to detect the association between cell type proportionality and cancer state. We detected several significant changes among the 25 cell clusters identified in PBMCs (Fig. 1B and Supp. Figure S1B). Overall, the cancer condition was characterised by increased numbers of dendritic cell types. The most associated cell types were plasmacytoid dendritic cells (pDCs) (FDR < 2.5 × 10^–4^), two cell clusters of conventional dendritic cells (cDC) (FDR = 5 × 10^–3^ and = 9.3 × 10^–2^) and regulatory T cells (Tregs) (FDR = 1 × 10^–3^) (Fig. 1B). On the contrary, cancer samples show marked depletion of B cells (memory, intermediate and immature; FDR = 1 × 10^–3^, = 8.8 × 10^–4^ and = 1 × 10^–2^) and CD8 + T cells (effector memory, FDR = 1.9 × 10^–2^). Unconventional T cell population MAIT (Mucosal-associated invariant T cells), known to kill tumour cells directly via MHC-independent mechanisms [49–51], was depleted in cancer samples (FDR = 2.9 × 10^–2^). Collectively, these results suggest that the blood of healthy donors and cancer patients contain distinct immune repertoires.

In addition to compositional changes, we observed significant (FDR < 0.05) transcriptional changes in cancer conditions (Fig. 1C and Supp. data file 1). Specifically, B cells show upregulation of HLA-DQA1, a gene related to antigen presentation. However, these cells showed decreased expression of inflammatory markers CD69, FOS and DUSP1 in cancer patients, thus indicating a less active inflammatory profile [52–55]. In contrast, the CD8 T and CD4 T (central and effector memory) cells show downregulation of interferon-induced transmembrane protein 1 (IFITM1), also known for its role in suppressing metastasis [56, 57]. In contrast, monocyte subsets are marked by high expression of immunomodulatory genes RORA, MYL9 and ITGB5 [52, 58–60]. Notably, XIST, a gene known for its role in X chromosome inactivation and its potential involvement in B cell function [61], was found to be upregulated in immature B cell and T cell populations. The monocytes, CD4 T, and CD8 T (central and effector memory) cells exhibit the most differentially expressed genes (Supp. Figure S1C). Overall, the systemic alterations observed in immune cell composition and phenotype among cancer patients may contribute to immune evasion by cancer cells and facilitate tumour progression.

### Relationship between circulating and tumour infiltrating immune cells

**Fig. 2:**
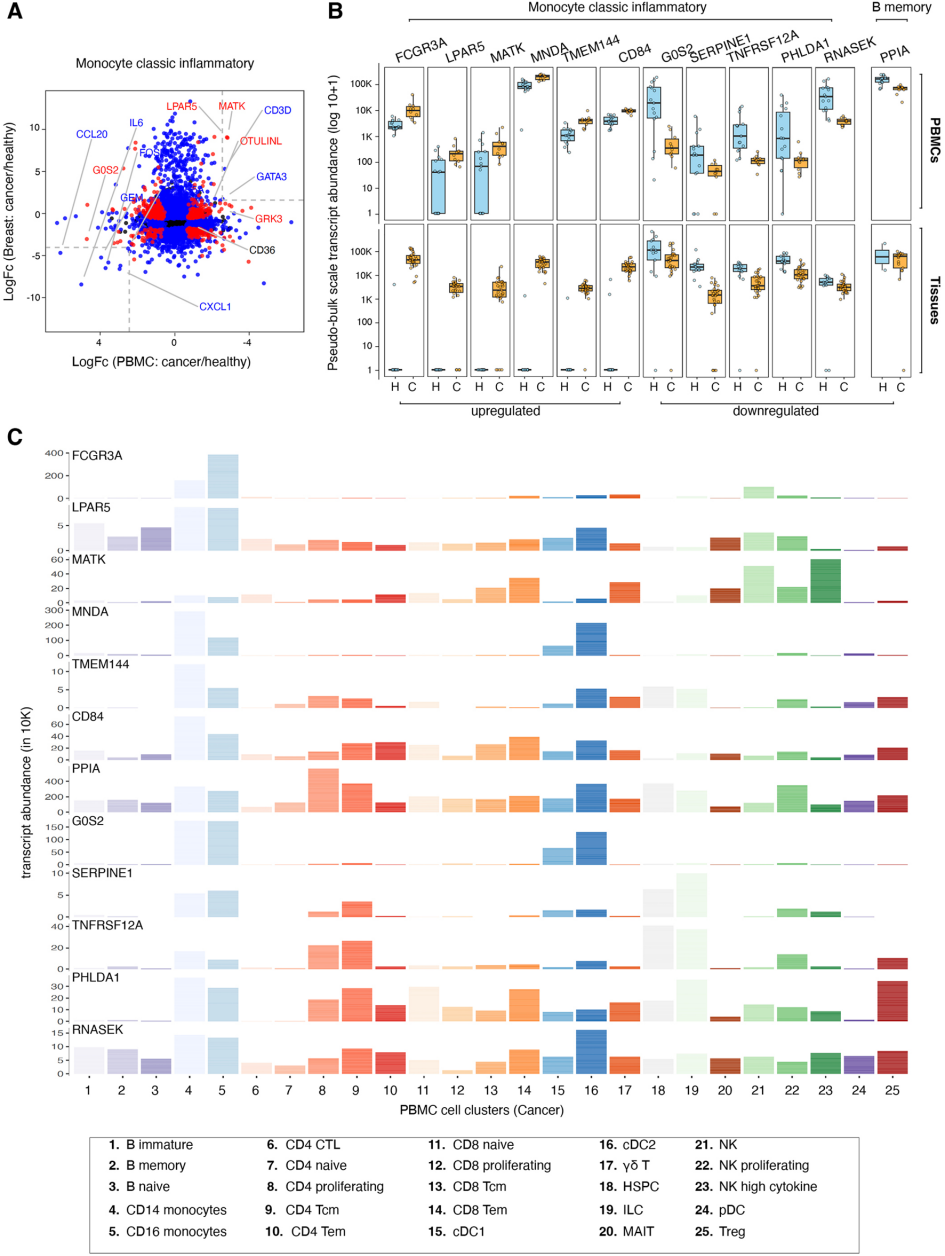
Integrated analysis of circulating and tumour infiltrating immune cells. **A** Gene expression changes (log fold-change) between PBMCs and breast tumour tissue across monocyte classic inflammatory cells. Genes are categorised by their significance in FDR (red: significant in both tissues, blue: significant in either tissue, black: not significant). Dashed lines draw thresholds for fold-change = 2.5 for reference, highlighting areas where genes exhibit substantial expression differences between the two conditions across tissues. **B** upper panel. Healthy/cancer (H/C) gene signature. Boxplots represent the scaled transcript abundance distribution of the cell-type/sample-specific pseudo-bulk data, healthy (blue) and cancer-associated (orange). **B** lower panel. Validation of the PBMC signature in breast tissue immune cells. **C** Transcript abundance per cell type (in cancer) for the 12-gene signature differentiating cancer from healthy state

The transcriptional reprogramming of circulating immune cells during disease progression might mirror the molecular changes in the corresponding cell types in the local tumour microenvironment (TME). To identify such cell-type-specific transcriptional changes, we integrated a single-cell atlas encompassing 48 breast tissue specimens (13 healthy breast tissues and 35 primary breast tumours [62]). The tumour profiles were selected to represent major breast cancer subtypes, including estrogen receptor (ER) + , HER2 + , and TNBC. We analysed the significantly differentially expressed PBMC genes (FDR < 0.05) in the corresponding cell types identified in the single-cell breast atlas (Supp. Figure S2 and Fig. 2A). The integrated transcriptome analysis revealed that inflammatory monocytes exhibited the most significant transcriptional changes between healthy and cancer states in PBMCs (Fig. 2B, top panel) and within the local tissue microenvironment (Fig. 2B, bottom panel). The top six upregulated genes are FCGR3A, LPAR5, MATK, MNDA, TMEM144 and CD84, while the top six downregulated genes are G0S2, SERPINE1, TNFRSF12A, PHLDA1, RNASEK and PPIA. Furthermore, expression analysis of these 12 genes in 25 immune cell clusters (Fig. 1B) confirmed their expression in monocytes (cell clusters # 4 and 5; Fig. 2C).

### Increasing metastatic burden is characterised by distinct changes in immune cell abundance and their transcriptomic profiles

Given that cellular and transcriptomic features of the peripheral immune system can distinguish metastatic from healthy states, we focused specifically on evaluating the molecular characteristics of immune cells in cancer samples to identify the more subtle but clinically significant distinction between low and high metastatic burden. Firstly, for cell annotation, we integrated the samples after QC and filtering using Seurat [32], identified cell clusters (Supp. Figure S3A) and manually annotated them against known lineage markers, with supporting evidence from the Azimuth mapping against the PBMC reference [31]. We identified major immune cell clusters corresponding to B cells, myeloid, plasmacytoid dendritic cells (pDC) and a lymphoid population of T cells and NK cells (Supp. Figure S3B-D).

The T/NK cell (#4) and myeloid cell clusters (#2) (Supp. Figure S3D) were independently re-clustered and re-analysed (Supp. Figure S4). For the T/NK cluster, sixteen transcriptional subclusters were identified in the lymphoid cluster, including CD4 T cells (six clusters), CD8 T cells (five clusters), NK cells (three clusters), gamma delta (γδ) T cells, and Mucosal-associated invariant T (MAIT) cells (Supp. Figure S4A). All cells transcribed varying levels of CD3G, a pan T-cell gene, whereas CD4 cells co-transcribed IL7R and KLRB1, while CD8 cells were marked by GNLY and GZMK (Supp. Figure S4B and C). The MAIT, NK and γδ T cells exclusively transcribed lineage markers NCR3, STMN1 and TRDC, respectively (Supp. Figure S4B and C). The in-depth investigation of the myeloid population revealed eight cell sub-clusters consisting of cDC2, monocyte (four clusters), myeloid migratory cells and two unexpected clusters of macrophage-like cells (Supp. Figure S4D). The cDC2 cells transcribe the high-affinity IgE receptor, FCER1A, while migratory myeloid cells co-transcribe high levels of chemokine PPBP (Supp. Figure S4E and F). The monocytes and myeloid migratory cells showed varying transcription of CD14, ITGAM and CD68 lineage markers. While the classical and non-classical monocytes could be distinguished with differential expression of CD14 and FCGR3A, both subsets surprisingly showed high abundance of inflammatory NK cell granule protein, NKG7 (Supp. Figure S4E and F).

**Fig. 3:**
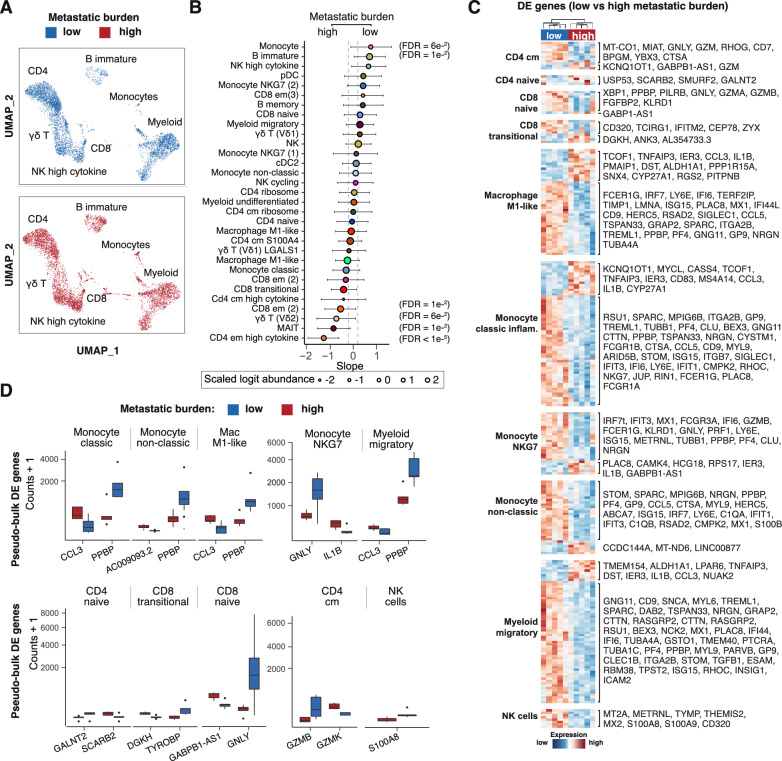
Metastatic burden redefines the cell composition and molecular characteristics of PBMCs. **A** Integrated UMAP plots showing single cells coloured by low (blue) and high (red) metastatic burden. **B** Differential cell composition analysis between low and high metastatic burden. Dots are coloured by cell type. Cell types enriched in low-metastatic-burden patients are at the top, while those enriched in high-metastatic-burden patients are at the bottom. The error bars represent the 95% credible interval for the estimates. The bold dot borders label the significantly enriched cell types (testing for an effect bigger than 0.2 logit fold change). **C** Scaled transcription abundance of cell-type-specific pseudo-bulk samples for the differentially abundant gene transcripts between low vs high metastasis burden. **D** Top difference per cell type. Boxplots showing the scaled transcript abundance distribution of the cell-type/sample-specific pseudo-bulk data for low (blue) or high (red) metastatic burden. For each cell type shown, the gene wise FDR values are: Monocytes (CCL3; FDR = 1e-^3^ and PPBP; FDR = 5e-^10^), Monocyte non-classic (AC009093.2; FDR = 7e-^6^ and PPBP; FDR = 3e-^5^), Macrophage M1-like (CCL3; FDR = 2e-^3^ and PPBP; FDR = 6e-^7^), Monocyte NKG7 (GNLY; FDR = 2e-^4^ and IL1B; FDR = 5e-^5^), Myeloid migratory (CCL3; FDR = 9e-^4^ and PPBP; FDR = 4e-^5^), CD4 naïve (GALANT2; FDR = 1e-^2^ and SCARB2; FDR = 1e-^2^), CD 8 T transitional (DGKH; FDR = 5e-^5^ and TYROBP; FDR = 2e-^5^), CD 8 T naïve (GABPB1-AS1; FDR = 7e-^3^ and GNLY; FDR = 4e-^3^), CD 4 Tcm (GZMB; FDR = 2e-^7^ and GZMK; FDR = 9e-^5^) and NK cells (S100A8; FDR = 1e-^3^)

Next, the cancer samples were dichotomised based on low (single organ; blue) and high (multiple organs; red) metastatic burden (Supp. table 1 and Fig. 3A). The PBMC composition analysis revealed differences associated with metastatic burden (Fig. 3B). Overall, the T cell compartment for CD4 + and CD8 + cells was significantly depleted in high-burden metastasis, including CD4 T effector memory and CD8 T effector memory cells (em; FDR < 1 × 10^–5^ and = 1 × 10^–2^). The relative depletion of unconventional T cells, including MAIT (FDR = 1 × 10^–2^) and gamma delta (γδ) T cells (Vδ 2 subset; FDR = 6 × 10^–2^), were among the strongest indicators of high-burden disease. In contrast, low-burden metastatic patients showed a systemic immune response skewed towards B cells (with immature being significant at FDR = 1 × 10^–2^) and monocytes (with a large proportional fold change of 1.8 and near significance FDR = 6 × 10^–2^). In the myeloid compartment, pre-dendritic, inflammatory classic monocytes and M1 macrophage-like cells were mildly depleted in the high-burden metastasis group. Collectively, these immune cell populations are key drivers of the anti-tumour immune response.

The enriched and depleted cell types also showed different transcriptional phenotypes between metastatic profiles (Fig. 3C). The most significant transcriptional differences were observed in CD4 T (central memory and naïve), CD8 + T (transitional and naïve), monocytes (classic, non-classic and NKG7 high), and migratory myeloid and macrophage M1-like cells (Fig. 3C). In high metastatic-burden disease, the CD4 T lymphoid populations show upregulation of GZMK (FDR 6 × 10^–2^; pro-inflammatory) [63] and downregulation of GZMB (FDR = 2 × 10^–7^; cytolytic activity) and CD8 T cells downregulate GNLY (FDR = 4 × 10^–3^; cytolytic activity) [64, 65] (Fig. 3D). However, NK cells in high metastatic disease are marked by relatively lower expression of S100A8 (FDR = 1 × 10^–2^; a pro-inflammatory factor that promotes metastasis) [66, 67]. In contrast, the myeloid cell types in high metastatic burden are marked by upregulation of CCL3 (FDR 1 × 10^–3^; acute inflammation) [68] and IL1B (FDR = 5 × 10^–5^; acute inflammatory response) [69, 70] and downregulation of PPBP (FDR = 4 × 10^–5^, glucose metabolism and plasminogen activator) [71] and GNLY (cytolytic activity) [65] (Fig. 3D). These findings describe the complex interplay of immune cell transcriptional activities in the context of metastatic cancer. The differential expression of key inflammatory and cytolytic genes in several immune cells highlights key pathways for immune response in high metastatic disease. Understanding these transcriptional changes is crucial for developing strategies to enhance anti-tumour immunity and potentially track metastasis.

### High metastatic burden disrupts key cell–cell communications in the circulating immune landscape

In the tissue microenvironment, intercellular crosstalk has been rigorously investigated [72–74]. Yet, the precise modalities governing communication among circulating immune cells in oncological contexts remain mostly unknown. To address this, we used CellChat [42] to investigate the co-transcriptional dynamics of ligand-receptor pairs across diverse cellular populations. Such exploration provides insights into both short (involving direct cell-to-cell contacts and matrix interactions) and long-range (mediated by secreted ligands) communication axes associated with metastatic burden.

**Fig. 4:**
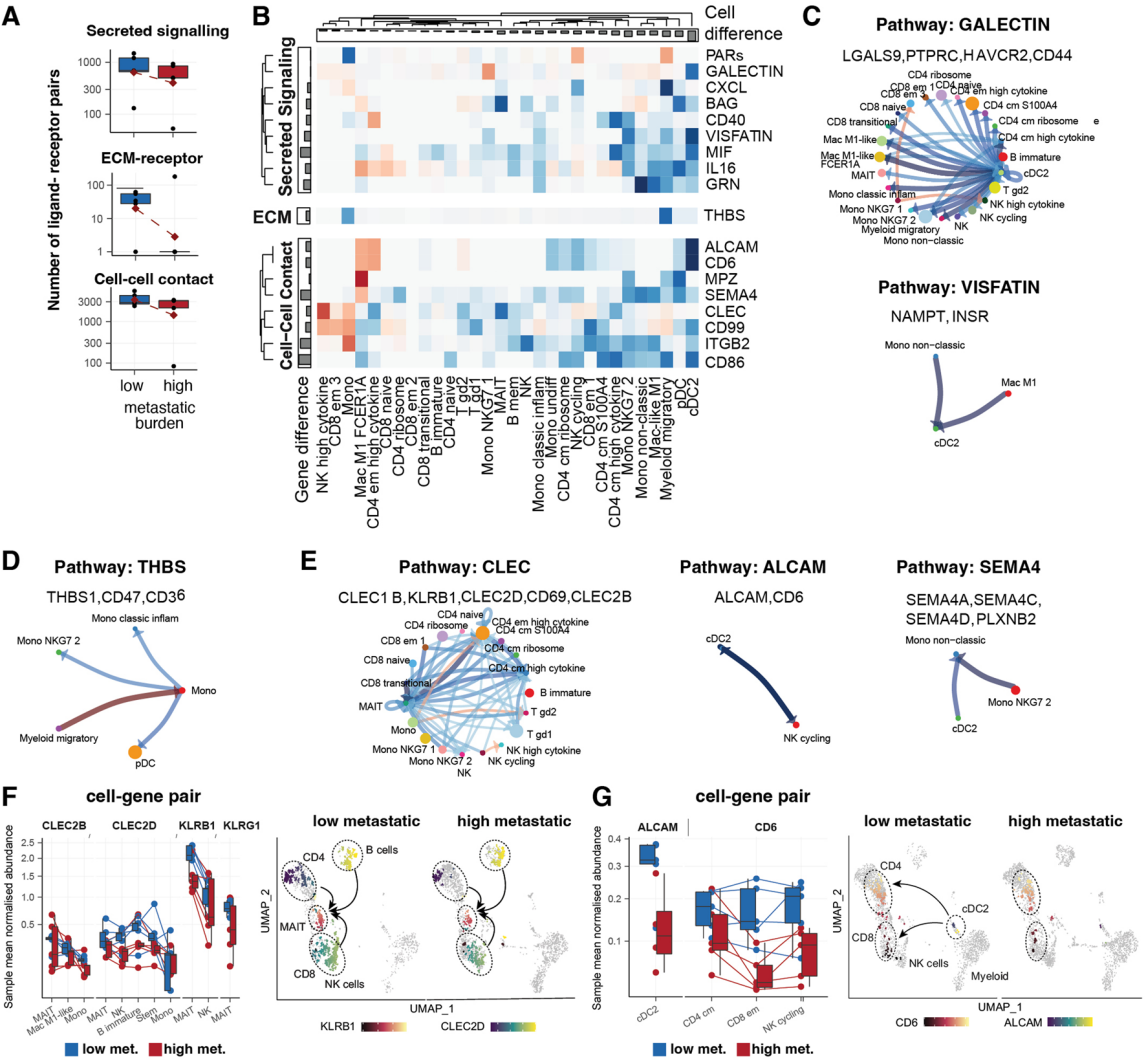
The extent of metastatic burden alters immune crosstalk profiles. **A** Comparison of the number of highly transcribed ligand-receptor pairs between PBMC samples from high and low metastatic burden patients. The communication axes are grouped by long-distance secreted signalling, extracellular matrix (ECM) receptors, and cell–cell interactions. The red diamond indicates the average count across samples. **B** Composite enrichment scores for cell-type/communication axes between a cell type (e.g. cDC2; x-axis) and all other cell types. Each communication axis can underlie several ligands and receptors. The colours represent the enrichment direction (blue for low and red for high metastatic burden disease). Communication axes are grouped by type. Bar plots represent the enrichment score average for cell types (columns) or communication axes (rows). **C**, **D**, and **E** Enrichment of communication among cell type pairs for the most differentially enriched communication axes (in either direction). The colours represent the enrichment direction (blue for low and red for high metastatic burden disease). The genes included in each communication axis are below the names of the communication axes. The arrows represent the direction of the communication (ligand outgoing, receptor incoming). Some communications are bi-directional. **F** Transcript abundance for the CLEC/KLRB1 ligand/receptor pair for the cell types that show enrichment. Colours represent conditions (blue for low and red for high metastatic burden). UMAP plot of cells coloured by transcription of either CLEC or KLRB1. The UMAP panels on the right side show the enrichment of communication between cell types for the most differentially enriched communication axes (in either direction). **G** Transcript abundance for the ALCAM/CD6 ligand/receptor pair for the cell types that show enrichment (dendritic cells with ligand and lymphocytes with the receptor). Colours represent conditions. UMAP plot of cells coloured by transcription of either ALCAM or CD6. The UMAP panels (right-side) show communication enrichment among cell types. The colours represent the enrichment direction (blue for low- and red for high-metastatic burden disease)

A pronounced decrease in intercellular communication, including short- and long-range signalling, was observed in high metastatic disease compared to low metastatic burden (Fig. 4A). Conventional dendritic cells (cDC2) showed the largest downregulation among all cell populations (Fig. 4B). The GALECTIN communication pathway was downregulated between cDC2 cells and the largest number of other cell types. This pathway includes LGALS9 as a ligand from dendritic cells and PTPRC, HAVCR2 and CD44 as receptors from target cell types (Fig. 4C). The Visfatin/NAMPT communication pathway was downregulated in fewer cell types, including inflammatory monocytes and dendritic cells. We also identified the downregulation of the secretory molecule Thrombospondin-1 (encoded by THBS) and its cognate receptors CD47 and CD36 [75] between monocytes and pDC2 for high metastatic burden (Fig. 4D).

Mucosal-associated invariant T cells (MAIT) showed several depleted communication axes, such as CD69, CLEC, and KLRB1 (Fig. 4E and F). For short-range interactions, the communication pathway ALCAM and CD6 showed the strongest downregulation in high-burden metastasis (Fig. 4E and G). Within this network, high cytokine-expressing CD4 T memory and γδ T cells emerge as key interactive hubs. Amongst myeloid cells, the SEM4A communication pathway, known to play a role in the T-cell priming [76], was significantly downregulated (FDR < 0.05) for high metastatic burden (Fig. 4E).

### Circulating unconventional T cell’s molecular profile changes with metastatic burden

Our study supports the importance of studying unconventional T cells in cancer metastasis and their potential to track disease progression. The pathogenic roles of MAIT and γδ T cells have been studied extensively [77, 78]. However, their role in breast cancer and metastasis is unclear. Interestingly, our PBMC analysis revealed depleted numbers of MAIT and γδ T cells in samples associated with high metastatic burden compared to healthy controls or low metastatic burden conditions (Figs. 1B and 3B). Hence, we re-clustered these cells from total PBMCs for further in-depth analysis.

In cancer samples, MAIT cells are marked by relatively higher expression of genes, including LTB, known to regulate proinflammatory response and development of tertiary lymphoid structures [79] and S100A4 involved in motility, recruitment and chemotaxis of inflammatory immune cells [80] (Supp. Figure S5A). In healthy samples, the MAIT cells show upregulation of NCR3 (cytotoxicity triggering receptor) [81], ARL4C (ADP-ribosylation factor-like protein associated with cancer progression) [82], and DUSP2 (a MAP kinase family member required for cellular differentiation and proliferation) [83] (Supp. Figure S5B). Moreover, the common genes between healthy and cancer MAIT cells include KLRB1, KLRG1 and GZMK (Supp. Figure S5C).

Compared to MAIT cells, the γδ T cells show distinct transcriptional heterogeneity in healthy and cancer conditions (Supp. Figure S6A and B). We interrogated re-clustered γδ T cells with previously described 30-gene signature [36] to identify Vδ1 and Vδ2 subsets in total γδ T cell population of healthy and cancer PBMCs (Supp. Figure S6A and B, left panels and S6C). As expected, Vδ1 and Vδ2 subsets express genes involved in T-cell mediated cytotoxicity (GNLY, GZMH, FGFBP2, GZMB, NKG7), immunosuppression (LGALS1) and, proinflammatory responses (GZMK, LTB, IL7R). Next, we determined the differentially expressed genes that revealed unique molecular differences within γδ T subsets (Supp. Figure S6A and B, right panels). Especially in cancer samples, Vδ1 subset express SH3BGRL3, CYBA, CCL5, CST7, TMSB10, FCGR3A, ZEB2, PRF1, IFITM2, HLA-E genes, whereas Vδ2 cells are marked by CD52, TPT1 and EEF1A1 genes.

**Fig. 5:**
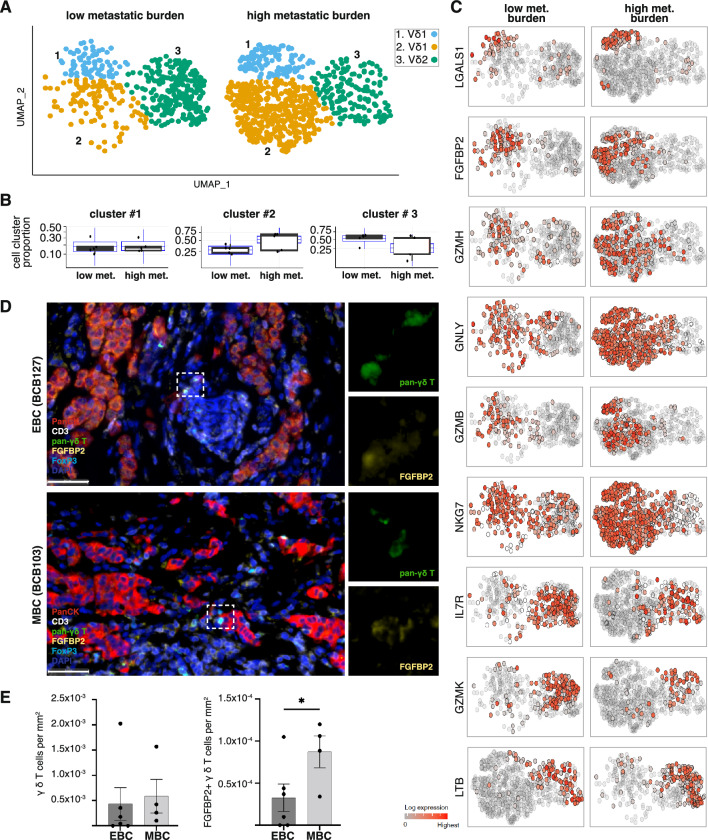
Gamma-delta T cells show transcriptional changes in metastatic patients with breast cancer. **A** UMAP plots of the re-clustered γδ T cells from low-metastatic (left panel) and high-metastatic burden samples (right panel). **B** Differential composition (estimated by sccomp) of the three gamma-delta cell clusters relative to the whole gamma-delta population between low and high-burden metastatic patients. Data points are samples. The blue boxplot represents the posterior predictive estimates that indicate the model’s descriptive accuracy for the data [37]. **C** UMAP plots of gamma-delta T cells in low-metastatic (left panel) and high-metastatic samples (right panel), coloured by the relative transcript abundance of the top 9 marker genes. **D** mIHC panel shows the expression of pan-cytokeratin (red; tumour cells), CD3 (white; T cells), pan-γδ T cell (green), FGFBP2 (yellow), FOXP3 (light blue) and DAPI (dark blue; nuclei) in primary tumour tissues from non-metastatic early breast cancer patients (EBC; top panel) and metastatic breast cancer patients (MBC; bottom panel). The white dotted box marks γδ T cells that co-express FGFBP2 and are shown in magnified images on the right side. **E** mIHC image quantification showing the total number of γδ T cells per mm^2^ of tissues (left panel) and FGFBP2 + γδ T cells (right panel) from EBC (n = 6) and MBC tumours (n = 4) with p-value (* = 0.0369)

To further delineate the γδ T cell phenotypic characteristics associated with metastasis, we interrogated the Vδ1 and Vδ2 subsets in the context of low and high metastatic burden cases (Fig. 5A). The Vδ1 cell cluster (2; yellow) was relatively enriched in PBMCs from patients with high metastatic burden compared to the Vδ2 cell cluster (3; green) (Fig. 5B). The Vδ2 cell cluster showed minor expression changes in proinflammatory genes IL7R, GZMK and LTB between low and high metastatic conditions (Fig. 5C). Whereas the Vδ1 subsets expressing cytolytic genes (FGFBP2, GNLY, GZMB, GZMH, LGALS1 and NKG7) were detected at increased frequency in patients with high metastatic burden (Fig. 5C). Therefore, we speculate that high-burden multi-organ metastasis drives phenotypic and compositional changes in circulating γδ T cell subsets. Whether metastasis orchestrates similar phenotypic changes in tissue-resident γδ T cells remains to be determined. To this end, we completed multiplex immunohistochemistry (mIHC) on primary tumour tissues from 6 non-metastatic early breast cancer (EBC) and 4 metastatic breast cancer (MBC) patients to determine the relative proportion of FGFBP2^+^ γδ T cells between these two conditions. The primary tumour tissues did not show significant changes in total γδ T cell numbers; however, the tissue-resident FGFBP2 + γδ T cells were significantly enriched in MBC tumours (Fig. 5D and E), suggesting that molecular mechanisms similar to the peripheral immune system may operate in the primary tumour microenvironment (TME) to regulate the recruitment of cytotoxic γδ T cell subsets.

## Discussion

**Fig. 6:**
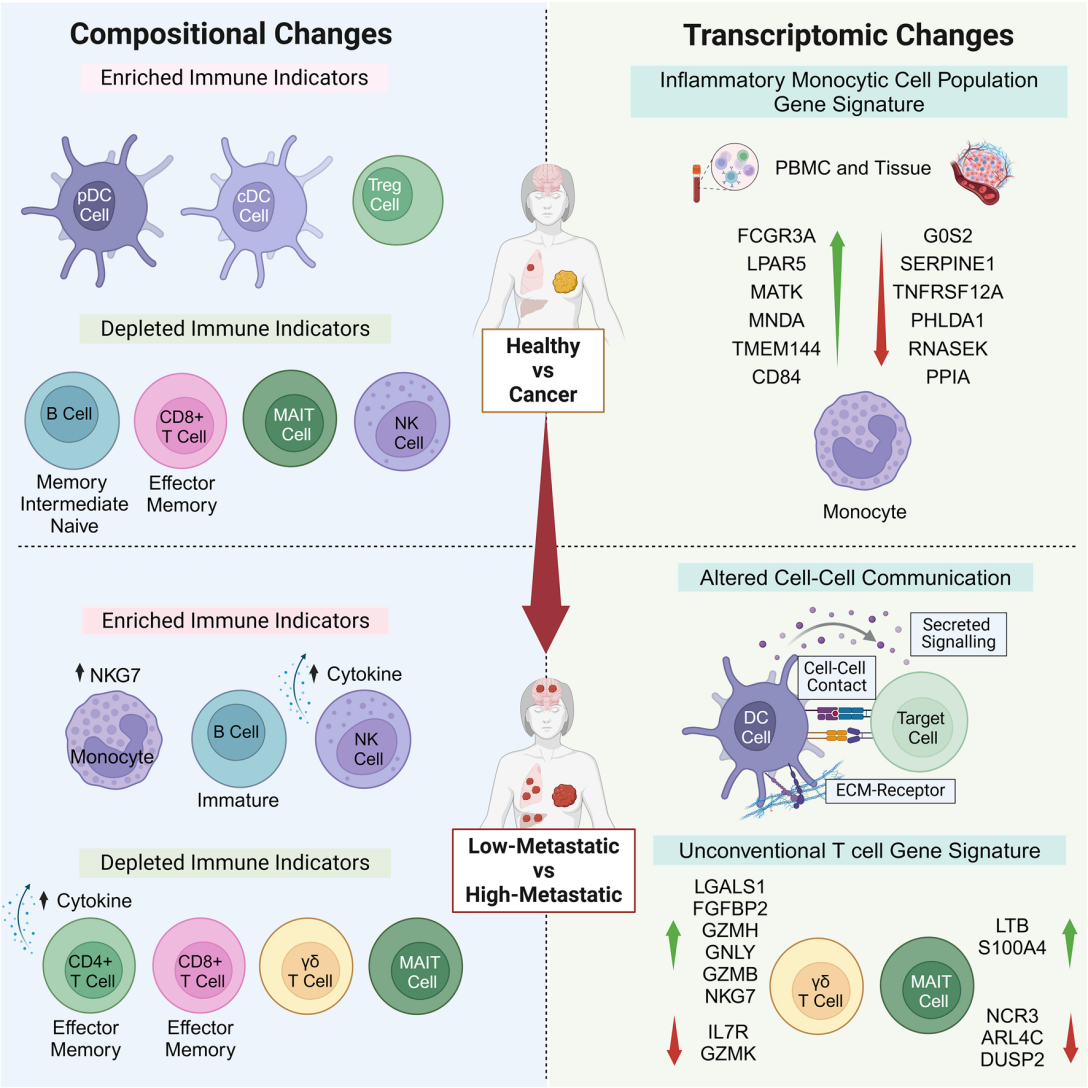
Schematic summary. The illustration summarises the compositional and transcriptomic changes in PBMCs between the healthy and cancer states (top half) and across different levels of metastatic burden (bottom half). Additionally, it highlights conserved transcriptional changes observed in monocytes from both PBMCs and breast tumors (top right)

Our study provides the first comprehensive analysis of single-cell transcriptomes of peripheral blood mononuclear cells (PBMCs) of breast cancer patients with metastatic disease (Fig. 6). Data analysis revealed four major cell clusters corresponding to B cells, myeloid, plasmacytoid dendritic cells (pDCs) and a lymphoid population comprising T and NK cells. A total of 30 transcriptional subclusters were identified in the myeloid and lymphoid cell compartments, including unconventional T cells. Interestingly, the classical and non-classical monocytes transcribe the inflammatory NK cell granule protein, NKG7. The NKG7 is required for NK and CD8^+^ T cell cytotoxic degranulation and CD4^+^ T cell activation and pro-inflammatory responses [84]. However, its functional relevance in monocyte activity needs further investigation.

Collectively, the composition and transcriptome analysis of PBMCs revealed that the blood of healthy donors and cancer patients contain distinct immune molecular repertoires. In the lymphoid compartment, cancer PBMCs exhibit substantial reductions in B cells (immature, intermediate and memory) and CD4 T and CD8 T cells (including central and effector memory phenotypes). In the myeloid compartment, metastatic breast cancer patients show enrichments of inflammatory monocytes characterised by high RORA, MYL9 and ITGB5 transcription [52, 58–60]. Furthermore, comparing single-cell transcriptome profiles of healthy individuals and those with metastatic breast cancer, we discovered various genes with differential expression in distinct circulating immune cell types. In essence, monocytes, CD4 T and CD8 T (central and effector memory) cells, displayed the majority of differentially expressed genes compared to other immune cell types. Overall, these systemic changes in immune cell composition and phenotype among cancer patients likely play an important role in metastatic progression.

Our study identified a unique 12-gene signature, hereinafter referred to as “breast cancer signature”, that seems to have a critical role in immune cell proliferation and inflammation. The six upregulated genes (FCGR3A, LPAR5, MATK, MNDA, TMEM144, and CD84) play key immunomodulatory roles in cancer progression and metastasis. LPAR5 promotes the progression of papillary thyroid carcinoma (PTC) [85], though its immune-related roles remain underexplored. MATK functions as a nonreceptor tyrosine kinase that negatively regulates Src family kinases and contributes to immune regulation [86], while MNDA is associated with myeloid cell function [87]. TMEM144, enriched in monocytes and dendritic cells, is linked to M2 macrophage-driven tumour progression [88], and CD84 serves as a surface marker for myeloid-derived suppressor cells (MDSCs) [89], facilitating their detection in cancer tissues. Conversely, the six downregulated genes (SERPINE1, TNFRSF12A, PHLDA1, RNASEK, PPIA, and G0S2) are involved in diverse cancer-related pathways. SERPINE1 is negatively associated with immune cells in colon adenocarcinoma [90], while TNFRSF12A (FN14a) drives tumour growth and metastasis [91, 92]. PHLDA1 downregulation predicts poor prognosis in breast cancer, especially in ER-negative patients [93]. RNASEK is linked to reduced prostate cancer risk [94], and PPIA acts as an oncogene in various cancers, negatively correlating with immune checkpoints and immune cell infiltration [95, 96]. The G0/G1 switch gene 2 (G0S2) has critical functions in cell survival and metastasis of cancer cells [97, 98] and in mediating a pro-inflammatory process by circulating monocytes [99]. These findings highlight the potential of these genes as biomarkers and therapeutic targets in cancer research. Furthermore, the “breast cancer signature” mainly consists of inflammatory monocyte-specific markers robustly translated in circulating PBMCs and within the primary breast tumours of all breast cancer subtypes [62]. The gene signature is mainly expressed by monocytic cells present in peripheral blood (Fig. 2), thus making it a suitable candidate for designing diagnostic strategies for metastatic disease. These results lay the groundwork for developing minimally invasive diagnostic tools and refining therapeutic approaches based on metastatic burden, but their practical application in clinical pathology will require additional research to streamline techniques and integrate findings into routine workflows. Additionally, further validation in larger cohorts is required to assess whether our findings hold across diverse populations.

The peripheral immunological changes are driven by or promote metastasis, remains to be resolved. A previous study utilising mass spectrometry-based proteomics has shown phenotypic similarities between PBMCs and the TME of matching breast cancer tissues [100], whereby the expression profiles of IL-17, PI3K-Akt, and components of HIF-1 signalling pathways were found to be conserved between circulating and tumour infiltrating immune cells. Further analysis comparing patients with metastatic disease revealed depletion of circulating CD4 and CD8 T effector memory cells, MAIT and γδ T cells in high metastatic burden cases. This pattern points toward possible unknown mechanisms operating in patients with low-burden metastatic disease that establish a more effective adaptive immune response and protection against the metastatic spread. The systemic immunity of low and high-burden disease is also characterised by distinct molecular changes in the lymphoid and myeloid populations, including monocytes, NK, and CD4 and CD8 T cells. Whether these phenotypic alterations in systemic immune cells and their compositional change are responsible for metastatic disease progression remains to be investigated.

Our data indicate that the circulating levels of unconventional T cells, including MAIT and γδ T cells, are depleted during cancer and disease progression. However, in high metastatic burden disease, the circulating Vδ1 population shows higher transcription of immunomodulatory and cytotoxicity genes NKG7, GZMH, GZMB, LGALS1 (Galectin 1) and FGFBP2 indicating a potential functional role of circulating γδ T cells during high burden metastatic disease. Interestingly, the total γδ T cell population is depleted in metastatic TME, pointing towards common mechanisms operating during metastasis that downregulate γδ T cells in the peripheral and tumour-local immune system. An integrated functional analysis of circulating and tumour resident γδ T subsets will be required to allow the design of efficient therapeutic strategies to boost γδ T cell activity against metastatic disease.

At the global transcriptome level, we observed downregulated cell–cell communication for high metastatic burden, especially between myeloid and lymphoid compartments. This included the short- (i.e., cell–cell contact, extracellular-matrix) and the long-range (secreted signalling) pathways. Key altered communication axes were ALCAM/CD6, between DC and T/NK cells, essential for dendritic/T cell effective interactions [101]; CD69, which is a hallmark of activated MAIT cells [102], which is also linked to the presence of CD25, the degranulation indicator CD107a, the generation of cytotoxic elements like perforin and granzyme B, and the emission of pro-inflammatory cytokines such as IFN-γ, TNF, IL-17, and CSF2/GM-CSF [102]; SEMA4/CD72, necessary for monocyte-promoted T cell activation [103]; and PECAM1 and ICAM, crucial to blood barrier migration [104]; and LCK, necessary for natural killer cell activation [105]. Additionally, key long-range immunomodulatory changes observed in lymphoid and myeloid compartments included the downregulation of cytokines (interferon-α/gamma and TNF-a pathways). Together, these findings uncover potential physiological functions of these phenotypic changes in circulating immune cells in metastatic disease and may provide a molecular basis of disease progression from low to high metastatic burden.

This study underscores the dynamic interplay between tumor-driven immune reprogramming and immune evasion mechanisms during metastatic progression. We propose that tumor cells reshape circulating immune profiles by promoting inflammatory monocytes while depleting critical immune subsets such as B cells, CD8 T and CD4 T cells, fostering a pro-tumor environment. Although γδ T cells and CD4 T cells express cytotoxic genes (e.g., GZMB, NKG7), their activity may be suppressed by tumor-induced immune checkpoint signaling and metabolic competition. Furthermore, disrupted myeloid-lymphoid interactions (e.g., ALCAM/CD6 axes) impair antigen presentation and T cell activation, facilitating immune escape. With increasing metastatic burden, chronic activation and exhaustion of CD4 T cells, along with effector memory cell depletion, further weaken adaptive immunity. These findings highlight the need for functional studies to clarify the causal links between peripheral immune alterations, immune evasion, and metastatic progression.

Our study is limited by its primary focus on metastatic burden, and therefore, further investigations into the role of tumour volume in influencing the immunological changes in the peripheral immune landscape are required. Additionally, different metastatic sites likely induce distinct immune responses due to variations in tissue microenvironments and organ-specific immune cell recruitment. These site-specific differences may affect peripheral immunity, potentially explaining differences in immune cell profiles between low metastatic burden and multi-site high metastatic burden cases observed in this study. Future research stratifying patients by metastatic site could clarify how these variations impact systemic immunity and metastatic progression. Integrating insights into metastatic burden, tumor volume, and site-specific immune responses is critical for advancing tailored immune-based diagnostics and therapies for metastatic breast cancer.

## Conclusions

Our data provide a strong rationale for using systemic immune cell composition and their marker genes to stratify metastatic breast cancer. In addition to circulating tumour DNA (ctDNA), PBMCs offer unique advantages in accessibility, sensitivity and real-time assessment of tumour dynamics. Unlike imaging techniques that may have limitations in detecting microscopic lesions [106–108], systemic PBMC composition and marker genes can offer a minimally invasive approach to capture comprehensive molecular information about tumours. While ctDNA analyses provide a snapshot of the mutational landscape of tumour cells, PBMC profiling allows for an indirect evaluation of the local immune system at both primary and metastatic sites. Therefore, PBMC-based biomarkers will facilitate timely diagnosis of breast cancer or disease relapse, and treatment response. We speculate that the findings from our study can be leveraged to study other metastatic cancers. Furthermore, therapeutic targeting of cell–cell communication pathways in PBMCs may also lead to new immunotherapy treatments against metastatic disease.

## Supplementary Information

Additional file1

Additional file2

## Data Availability

Sequence data have been deposited in the GEO database under accession code GSE291055 and the codes used for the analysis have been deposited in https://github.com/stemangiola/mangiola_et_al_2025_breast_cancer_pbmc.
