# Supplementary material for: Circulating immune cells exhibit distinct traits linked to metastatic burden in breast cancer
Source: Breast Cancer Res. 2025 May 8;27:73. doi: 10.1186/s13058-025-01982-2 (PMC12063295; doi:10.1186/s13058-025-01982-2)
Supplement: Supplementary file 2 — Additional file2 [file 13058_2025_1982_MOESM2_ESM.pdf]

Supplementary Materials for

Circulating immune cells exhibit distinct traits linked to metastatic burden in breast cancer

Mangiola et al

*Primary corresponding author. Email: bhupinder.pal@onjcri.org.au

This PDF file includes:

Figs. S1 to S6
Tables S1 to S3

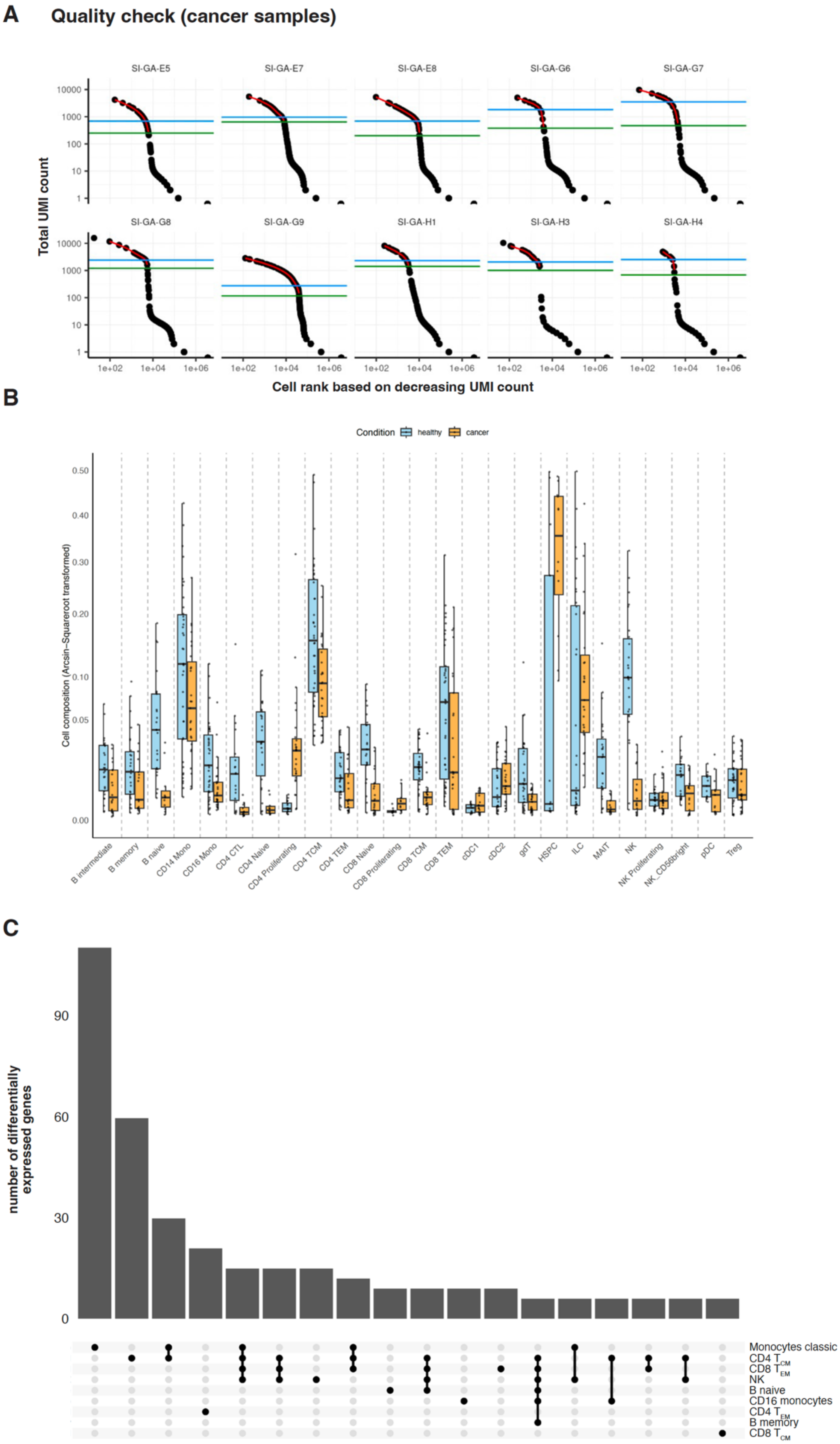
A
Quality check (cancer samples)
SI-GA-E5
SI-GA-E7
SI-GA-E8
SI-GA-G6
SI-GA-G7
SI-GA-G8
SI-GA-G9
SI-GA-H1
SI-GA-H3
SI-GA-H4
Total UMI count
Cell rank based on decreasing UMI count
B
Condition healthy cancer
Cell composition (Arcsin-Squareroot transformed)
B intermediate
B memory
B naive
CD14 Mono
CD16 Mono
CD4 CTL
CD4 Naive
CD4 Proliferating
CD4 TCM
CD4 TEM
CD8 Naive
CD8 Proliferating
CD8 TCM
CD8 TEM
cDC1
cDC2
gdT
HSPC
ILC
MAIT
NK
NK Proliferating
NK_CD56bright
pDC
Treg
C
number of differentially expressed genes
Monocytes classic
CD4 T_{CM}
CD8 T_{EM}
NK
B naive
CD16 monocytes
CD4 T_{EM}
B memory
CD8 T_{CM}

Fig. S1. Quality check analysis of healthy and cancer single-cell transcriptome profiles. A. Ranks of cells by their total RNA counts. These plots were used to detect empty droplets according to the green inflection line. **B.** Cell type proportionality for the cell types ranked by their differential abundance. The red triangles represent outliers identified by sccomp. **C.** UpSet plot illustrating the number of differentially expressed genes across various immune cell types. The bar chart at the top displays the total number of differentially expressed genes for each cell type, while the dot matrix below indicates the intersections of differentially expressed genes among the cell types. The cell types include monocytes classic, CD4 Tcm, CD8 Tem, NK, B naïve, Cd16 monocytes, CD4 Tem, B memory and CD 8 Tcm. The intersecting lines represent shared differentially expressed genes between the respective cell types.

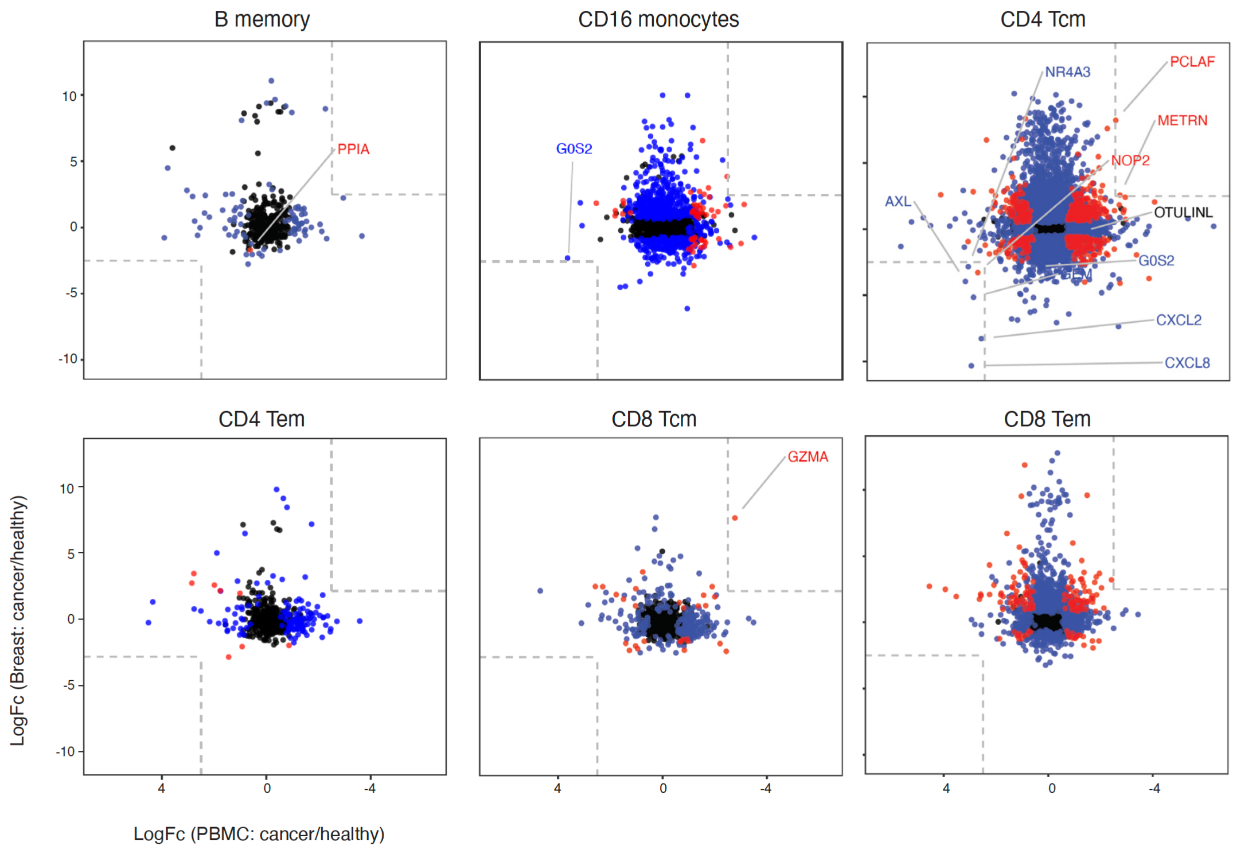

Fig. S2. Integrated analysis of single cell RNAseq profile of PBMCs and breast tissue (normal and cancer). Gene expression changes (log fold-change) between peripheral blood mononuclear cells (PBMCs) and breast tumour tissue across different immune cell types. Genes are categorised by their significance in FDR (red: significant in both tissues, blue: significant in either tissue, black: not significant). Dashed lines draw thresholds for fold-change = 2.5 for reference, highlighting areas where genes exhibit substantial expression differences between the two conditions across tissues.

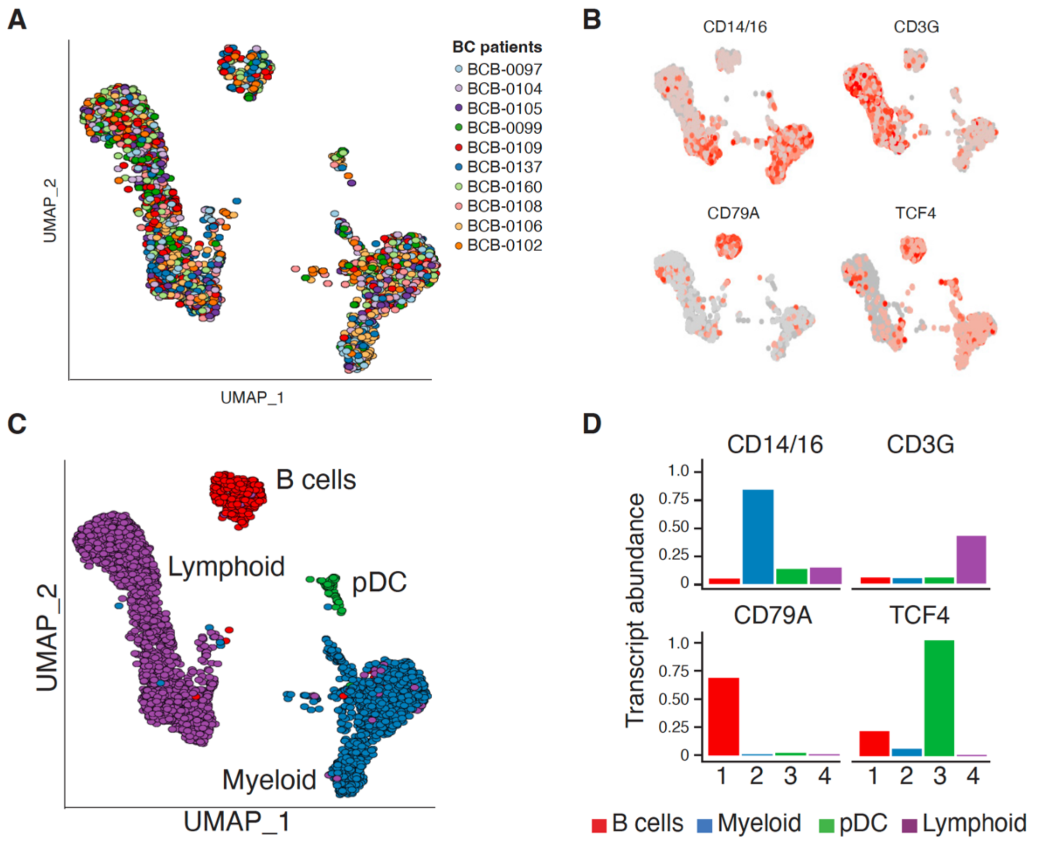

Fig. S3. Composition and transcriptome analysis of circulating immune cells in breast cancer patients. A. UMAP plot coloured by BC patient sample. **B.** UMAP plot coloured by transcript abundance of the marker genes for the four major cell types (B, T+NK, monocyte-derived, and plasmacytoid dendritic cells). **C.** UMAP plot coloured by four major cell types (B, T+NK, monocyte-derived, and plasmacytoid dendritic cells). **D.** Summary of the scaled relative transcript abundance of the marker genes for the four major cell types.

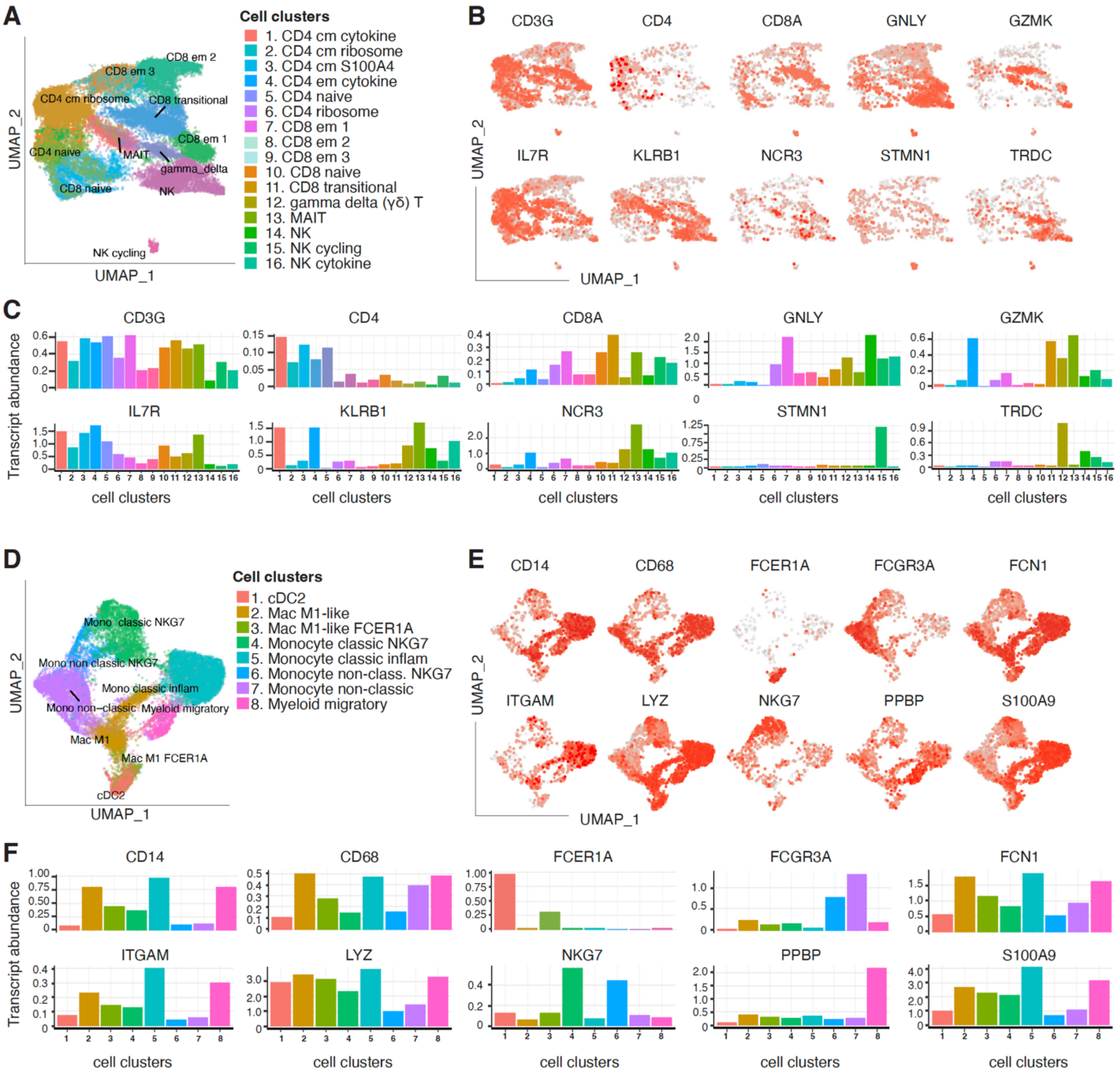

Fig. S4. Mapping cellular heterogeneity in myeloid and lymphoid cell compartments of PBMCs in metastatic breast cancer patients. A. UMAP plot of the lymphocytes re-analysed in isolation, coloured by cell type. **B.** UMAP plots coloured by the relative transcript abundance of marker genes for the lymphocyte cell types. **C.** Summary of the scaled relative transcript abundance of the lymphocyte marker genes. **D.** UMAP plot of the myeloid cells re-analysed in isolation, coloured by cell type. **E.** UMAP plots coloured by the relative transcript abundance of marker genes for the myeloid cells. **F.** Summary of the scaled relative transcript abundance of the marker genes for the myeloid cells.

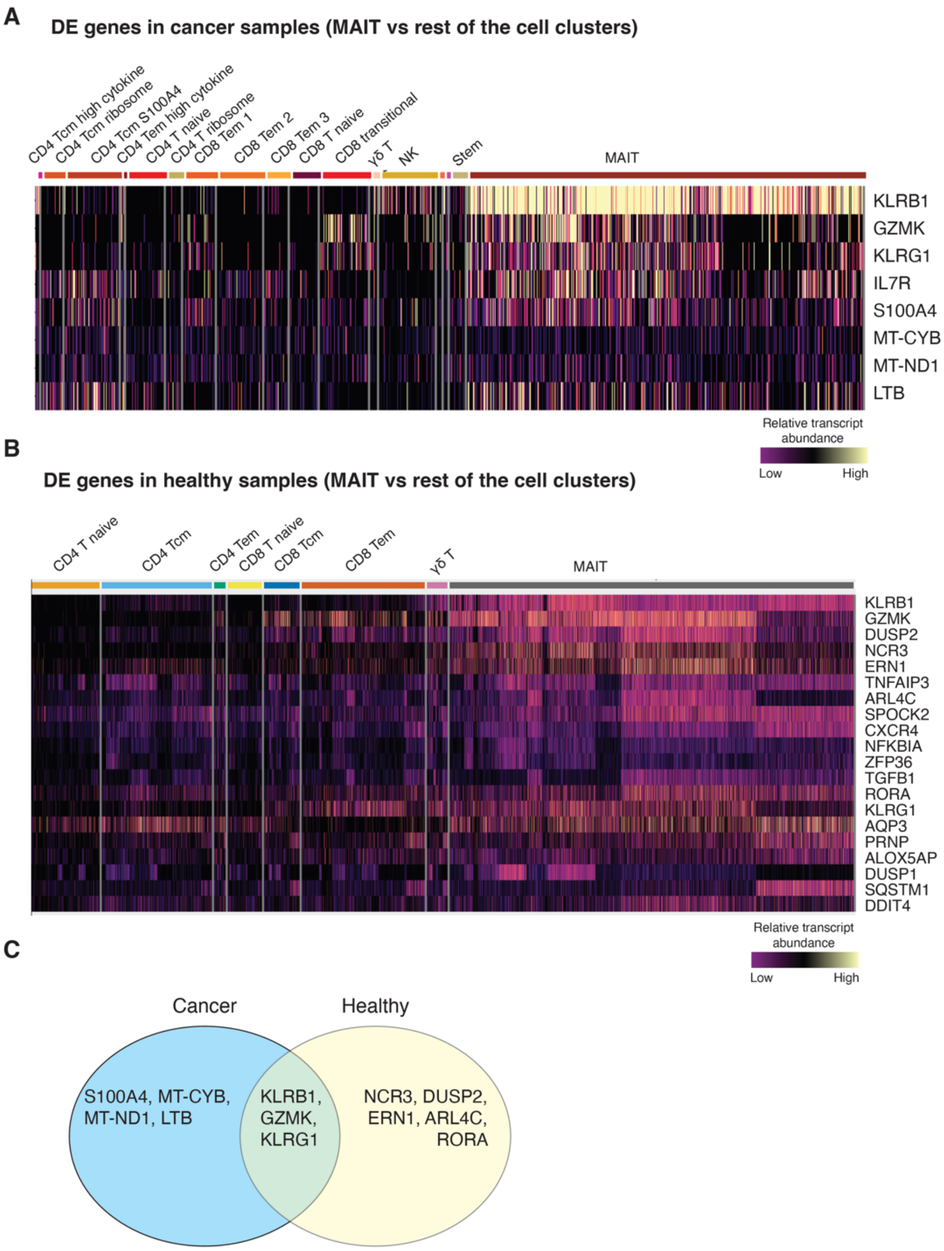

Fig. S5. Gene expression analysis of circulating MAIT cells in healthy and breast cancer samples. A-B. Heat map showing top differentially abundant gene transcripts for MAIT cells compared to other lymphocytes. MAIT cells were re-clustered from healthy and cancer samples analysed in Figure 1. The heatmap is coloured by the row-scaled relative abundance of marker-gene transcripts. **C.** Top marker genes of MAIT cells in cancer and healthy cohorts.

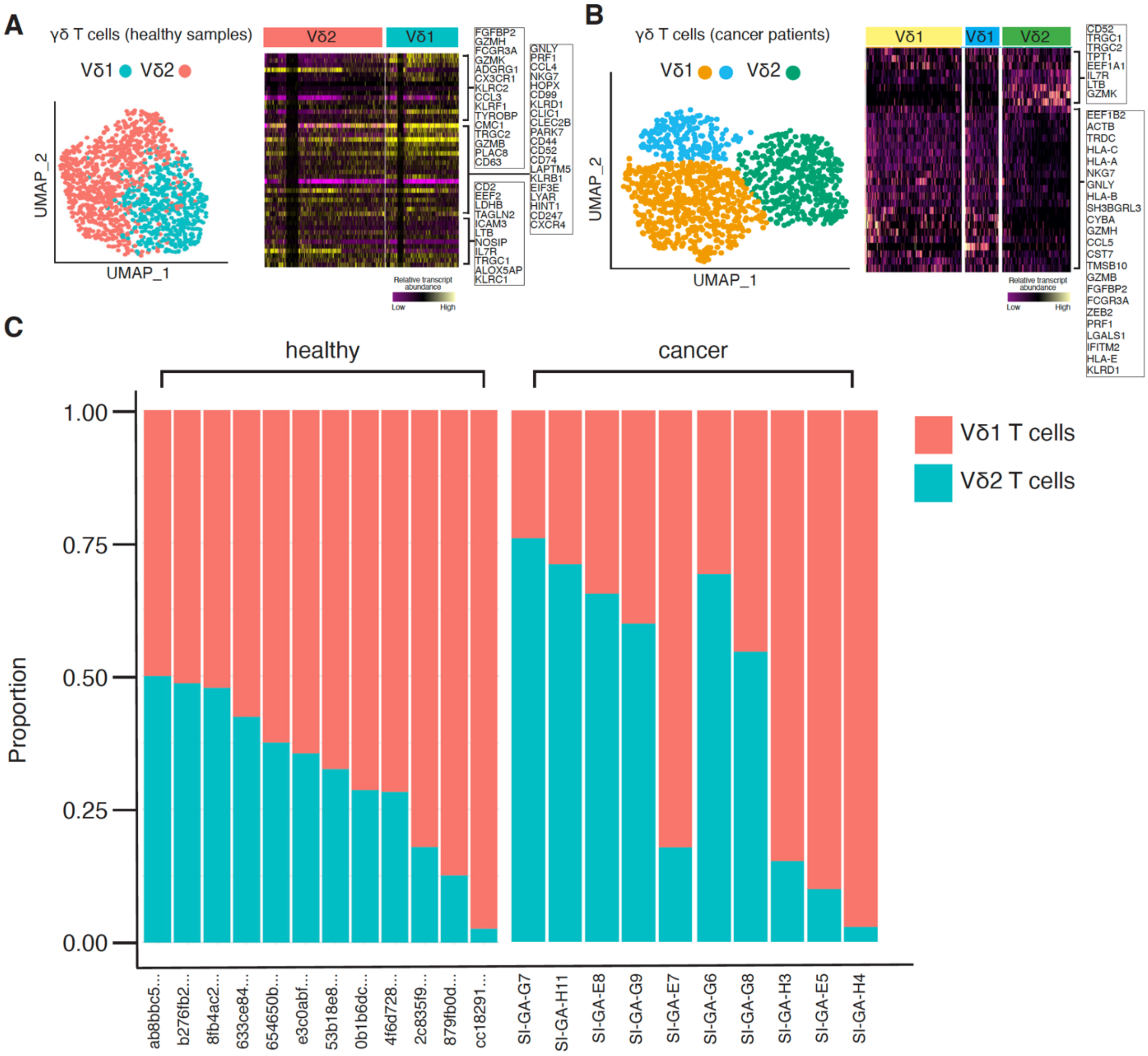

Fig. S6. Circulating Vδ 1 and Vδ 2 subsets show phenotypic changes in metastatic breast cancer. A-B. UMAP plots of the re-clustered γδ T cells from PBMCs of healthy and metastatic breast cancer cohorts, coloured by the relative transcript abundance of the top marker genes. The heatmaps show top differentially abundant transcripts in γδ T subsets in healthy (**A**) and cancer (**B**) PBMC samples. Heatmaps are coloured by the relative abundance of marker-gene transcripts (row-scaled). **C.** The plot shows the relative proportion of Vδ 1 and Vδ 2 T cell subtypes in healthy and metastatic cancer patients (including low metastatic burden cohort).

Table S1. Samples used for single cell transcriptome analysis

Sample		Age at sample collection	Breast cancer subtype	Metastatic burden	Metastatic site(s)	Systemic therapy	
label	type					before metastatic diagnosis	for metastatic disease and given at the time of sample collection
BCB-0097	Cancer	39	Luminal B	Low	liver	AC	Nab-paclitaxel
BCB-0099	Cancer	47	Luminal - > TNBC	Low	liver	AC-T + Tam	N/A
BCB-0109	Cancer	41	HER2+	Low	liver	AC + H + Tam	T + HP
BCB-0104	Cancer	51	Her2+ - >TNBC	Low	skin	N/A	AC (1 cycle),<br>D + HP,<br>capecitabine, liposomal-doxorubucin, Eribulin
BCB-0105	Cancer	48	Luminal B	Low	mediastinal lymph node (LN)	AC-T + AI	Ribociclib + AI
BCB-0137	Cancer	61	Her2+	high	Axilla, SC LNs, soft tissues	AC-T + H	D + HP,<br>T-DM1
BCB-0160	Cancer	61	Her2+	high	clivia, femur, thoracic and lumbar spine, ribs	AC-T + H + AI	T + HP,<br>HP + AI + Ramipril (ACE inhibitor)
BCB-0108	Cancer	57	TNBC	high	Multiple subcutaneous skin lesions, Pleura, Peritoneum/Omentum	AC-T	capecitabine, eribulin, liposomal-doxorubucin, anti-CD40 immunotherapy (trial),<br>carboplatin, vinorelbine
BCB-0106	Cancer	69	Luminal A	high	Axilla, lungs, Liver, Bone, Pleura, Skin	N/A	Ribociclib + AI
BCB-0102	Cancer	52	Luminal B	high	Liver, Bone (multiple, spine, femur, ribs)	FEC-D, Tam, goserelin + AI	Fulvestrant + BET inhibitor (trial), BCL2-inhibitor + Tam (trial), Capecitabine

Sample	Type	Age at sample collection	Breast cancer subtype	Metastatic burden	Metastatic site(s)	Systemic therapy
SCP345_860	Healthy	Not available	Not applicable	Not applicable	Not applicable	Not applicable
SCP424_pbmc2	Healthy	Not available				
SRR7244582	Healthy	Not available				
0b1b6dc431a59a8e4e773cd6f7c4cb54	Healthy	55				
ab8bbc52df887a48b399ce3cbc485aa5	Healthy	40				
8fb4ac29d2658fd16aac387429bb226d	Healthy	54				
b276fb26d57433c65134feae54e83400	Healthy	69				
e3c0abfd555e96869118d756c58ff614	Healthy	53				
2c835f96b27ecbf0802cd11154262157	Healthy	62				
654650b796a9d73bf68f79c7f9fdc3e6	Healthy	54				
879fb0d595b4fdf5fe8fb56f42dc66ee	Healthy	44				
4f6d728ce5ea9fb3b141285ddd4ea593	Healthy	64				
633ce84b0adb6fd779ecb8e4ce90d1fc	Healthy	54				
53b18e8f9b9476afa8e43073241e80b1	Healthy	40				
cc1829113fa8da8fed5f03c9fc7a193d___cv19_0711_ln2_samples_1_3	Healthy	54				

AC – Doxorubicin + Cyclophosphamide
T – Paclitaxel
D - Docetaxel
H – Trastuzumab
P - Pertuzumab
Tam – Tamoxifen
AI – Aromatase Inhibitor

Table S2. Single cell transcriptome quality check

Sample label	Sample type	Sum counts	Median counts	Median features
BCB-0097	Cancer	6977726	1326	705
BCB-0099	Cancer	9477448	2566.5	1040.5
BCB-0109	Cancer	20105016	3188	1155
BCB-0104	Cancer	8703971	2826	1152
BCB-0105	Cancer	12594050	3637	1377
BCB-0137	Cancer	13428707	1342	688
BCB-0160	Cancer	11045516	1069	500
BCB-0108	Cancer	9686781	3299.5	1223.5
BCB-0106	Cancer	16769560	629	364
BCB-0102	Cancer	10150130	4109.5	1384

Table S3. Breast tumour tissue sample details

Sample label	Sample type	Age	Tumour subtype	Disease stage	Tumour site(s)	Treatment
BCB127	Cancer	34	TNBC	EBC	primary	Untreated
BCB151	Cancer	67	Luminal A	EBC	primary	Untreated
BCB186	Cancer	55	Her2+	EBC	primary	Untreated
SCG013	Cancer	83	Luminal A	EBC	primary	Untreated
SCG023	Cancer	35	TNBC	EBC	primary	AC-T
SCG024	Cancer	70	Luminal A	EBC	primary	Untreated
SCG016	Cancer	45	Luminal B	MBC	primary	Untreated
BCB188	Cancer	27	Her2+	MBC	primary	Untreated
BCB103	Cancer	63	Her2+	MBC	primary	Untreated
SCG027	Cancer	33	TNBC	MBC	primary	Untreated

EBC = early breast cancer, MBC = metastatic breast cancer
